# Information Behaviour and Knowledge of Patients Before Radical Prostatectomy

**DOI:** 10.3390/cancers17020300

**Published:** 2025-01-17

**Authors:** Christopher Hirtsiefer, Anna Vogelgesang, Fabian Falkenbach, Mona Kafka, Annemarie Uhlig, Tim Nestler, Cem Aksoy, Iva Simunovic, Johannes Huber, Isabel Heidegger, Markus Graefen, Marianne Leitsmann, Christian Thomas, Martin Baunacke

**Affiliations:** 1 Klinik und Poliklinik für Urologie, Universitätsklinikum Carl Gustav Carus Dresden, 01307 Dresden, Germany; christopher.hirtsiefer@ukdd.de (C.H.); anna.vogelgesang@ukdd.de (A.V.); christian.thomas@ukdd.de (C.T.); 2 Martini-Klinik Prostatakarzinomzentrum, Universitätsklinikum Hamburg-Eppendorf, 20251 Hamburg, Germany; f.falkenbach@uke.de (F.F.); graefen@uke.de (M.G.); 3 Universitätsklinik für Urologie, Medizinische Universität Innsbruck, 6020 Innsbruck, Austria; mona.kafka@i-med.ac.at (M.K.); isabel-maria.heidegger@i-med.ac.at (I.H.); 4 Klinik für Urologie, Universitätsmedizin Göttingen, 37075 Göttingen, Germany; annemarie.uhlig@med.uni-goettingen.de; 5 Klinik für Urologie, Bundeswehrzentralrankenhaus Koblenz, 56072 Koblenz, Germany; timnestler@bundeswehr.org; 6 Klinik für Urologie, Universitätsklinikum Gießen und Marburg, 35043 Marburg, Germany; cem.aksoy@uni-marburg.de (C.A.); johannes.huber@uni-marburg.de (J.H.); 7 Universitätsklinik für Urologie, Medizinische Universität Graz, 8010 Graz, Austria; iva.simunovic@medunigraz.at (I.S.); marianne.leitsmann@medunigraz.at (M.L.); 8 Institute for Applied Quality Improvement and Research in Health Care, 37073 Göttingen, Germany

**Keywords:** radical prostatectomy, robot-assisted surgery, patient decision-making, informational behaviour, prostate cancer treatment, RARP, ORP

## Abstract

Background/Objectives: Robot-assisted and open radical prostatectomy (RARP and ORP) are established procedures for localized prostate cancer, with comparable oncological and functional outcomes. Little is known about patients’ knowledge of both procedures. This study aimed to examine comparatively the informational behaviour and knowledge of patients undergoing ORP vs. RARP. Methods: This prospective, multicentre study included patients who underwent RARP or ORP prior to presurgery counselling. The questionnaires gathered information about patients’ information-seeking behaviours and their assessment of outcomes for RARP vs. ORP. We investigated risk factors for the misperception of procedure outcomes. Results: A total of 508 patients were included (307 RARP (60%); 201 ORP (40%)). The most common sources of information were outpatient urologists (84%), urologic departments (67%) and the internet (57%). Compared with ORP, RARP patients more often received the same amount of information about both procedures (60% vs. 40%, *p* < 0.001). Compared with ORP, RARP patients wrongfully considered their procedure to be superior in terms of oncological and functional outcomes. In the multivariable analysis, age > 66 years (OR 2.1, *p* = 0.02), no high school degree (OR 1.9, *p* = 0.04), unbalanced information search (OR 2.4, *p* = 0.02), RARP patient status (OR 8.9, *p* < 0.001), and treatment at a centre offering only one procedure (OR 3.5, *p* < 0.001) were independent predictors of misperception. Conclusions: RARP patients wrongfully considered their intervention to be oncologically and functionally more beneficial than ORP patients perceived it to be. This may be due to unbalanced sources of information. Urologists and surgical centres must address this misperception to enable patients to make informed decisions.

## Simple Summary

This study evaluated how patients preparing for radical prostatectomy gather information and make decisions. This study is based on surveys handed out at seven urological centres in Germany and Austria prior to presurgery counselling. Patients planning for robot-assisted prostatectomy often believe that their procedure offers better outcomes, which is not clearly backed by scientific evidence. This finding is interesting, as those same patients gather more balanced information than those planned for open prostatectomy. Urologists and surgical centres remain the primary information sources, although the internet plays a significant role. This study underscores the importance of providing balanced, in-person information, as higher age, lower education, unbalanced information behaviour and the available operative options at the urological centre are predictors of misconceptions on operative outcomes of prostatectomy.

## 1. Introduction

Patients with locally confined stages (cT1-2/cN0/cM0) of prostate cancer (PCa) are candidates for radical prostatectomy (RP) with curative intent. Since the introduction of robot-assisted radical prostatectomy (RARP) in 2001, this procedure has become one of the most frequently performed operations in robot-assisted surgery [1,2]. Nevertheless, open RP (ORP) remained a well-established and widely used alternative.

The main aspects of comparison between the two procedures include hospitalization, procedure duration, intraoperative and postoperative complication rates, recovery, costs and, lastly, functional and oncological outcomes. RARP has clear advantages in certain aspects, such as less intraoperative blood loss, faster wound healing and shorter index hospitalisation [3,4,5]. RARP superiority in terms of postoperative complications is inconsistently reported but assumable [3,4,6,7,8].

Disadvantages of RARP are especially the longer procedure duration and higher cost of index hospitalization [6,8], which is a reason why some health care systems continue to rely (also) on ORP.

Despite the higher cost of index hospitalization, it was demonstrated that the total cumulative cost, including insurers’ and patients’ costs for inpatient and outpatient services, as well as lost working hours is ultimately comparable for RARP and ORP [6]. This stems from shorter hospitalization and lower readmission and complication rates in RARP [6].

Nevertheless, RARP has not superseded ORP, as both techniques continue to be evaluated in terms of oncological and functional efficacy, patient satisfaction, and efficiency.

Previous studies have shown long-term indifference concerning oncologic performance and postoperative continence [5,9,10,11,12]. The nerve-sparing technique [13] allows for the preservation of erectile function in both procedures. A number of short-term studies have indicated that RARP may offer superior outcomes in terms of postoperative erectile function. A meta-analysis yielded contradictory results, indicating functional equivalence at 24 months [8]. Prospective, multicentre studies with longer follow-up periods corroborated these findings [10,14].

There is an increasing trend towards robot-assisted prostatectomy in numerous countries. The prevalence of robot-assisted prostatectomy varies considerably between countries, with some health care systems having adopted the technology almost entirely, whereas in others, patients have to address the fact that there are clinics that offer only one procedure or both procedures [15]. It has been demonstrated that RARP has an attractive appeal [16]. A study conducted in England showed the impact of the introduction of RARP on the number of cases in hospitals with and without robots. There was a decrease in RP in hospitals without a robot-assisted system [17]. This prompts the question of the extent to which these developments are driven not only by purely medical considerations but also by a distorted media portrayal [18].

There is a paucity of knowledge regarding the informative behaviour of PCa patients before they choose a prostatectomy approach. In general, 56% of the European population turn to the internet for health information [19]. Online information about RP is skewed towards RARP, with a higher prevalence of RARP-related videos and patient-oriented websites. There is hardly any information about ORP on the internet [18,20]. The aim of this study was to investigate which sources patients use to find information about ORP and RARP, their lay assessment of both procedures and which risk factors lead to misperception of advantageous outcomes.

## 2. Materials and Methods

This prospective, multicentric observational study was conducted across seven urologic departments in Germany and Austria between January 2022 and December 2023. Four centres offered both ORP and RARP, two offered ORP and one offered RARP exclusively. Ethics committee approval was obtained in advance by the ethics committee of the Technical University of Dresden (BO-EK-438092021), as well as by local ethic committees where needed (Ethics Committee of University Medicine Göttingen, 11/2/22 Ü, 24 March 2022; Ethics Committee of the Medical Association of Rhineland-Palatinate/Koblenz, 2022-16322-andere Forschung, 11 March 2022). All study participants provided informed consent.

Patients scheduled for either RARP or ORP completed a questionnaire prior to their presurgery consultation and one year later. The current work evaluates the preoperative questionnaire. The questionnaire asked for demographic information (educational degree, household net income), information on the referring urologist, decision behaviour (in general, concerning surgical technique and choice of urologic centre), informative behaviour (information sources used, sources regarded important, internet usage, second opinion offers, usage of second opinion) and comparative perceptions of the risks and advantages of both techniques.

Informative behaviour involves the use of information sources. Patients were asked to rank the importance of information sources on a Likert scale. Online information behaviour was further explored by assessing weekly internet usage for health-related issues, followed by a multiple-choice question on the sources used. Patients were asked whether they had mainly received information on one of the two procedures in their search for information or whether they had received a balance of information on both procedures. Decision behaviour was determined by the validated Control Preference Scale for health-related decisions in general, as well as for decisions regarding RARP vs. ORP and the selection of the urologic centre performing the RP [21].

The perceptions of oncological outcome, functional outcome and further postsurgical aspects were evaluated comparatively by asking patients to estimate on a Likert scale if the ORP or RARP performed better.

To determine risk factors for false lay knowledge, a statement on the oncological outcomes of the two surgical procedures was taken as a starting point. We chose the oncological outcome for this purpose, since it is crucial for the patient, and the results showed no difference between the two surgical procedures [8,12]. The patients who stated that one of the two procedures was superior in terms of oncological outcome were defined as “unknowing”. Patients who indicated no difference were defined as “knowledgeable”. The study centres provided medical information (tumour classification, Gleason grade, PSA value, D’Amico risk group and planned surgical technique).

The data were analysed via the chi^2^ test and *t*-tests. Logistic regression models were used for the multivariable analyses. The parameters with statistical significance in the univariate analysis were included in the logistic regression models, along with the decision-making for empirical reasons. For age, the median was set as the cut-off. *p* < 0.05 was considered to indicate significance. All calculations were performed using “IBM SPSS Statistics 29” (Armonk, NY, USA).

## 3. Results

### 3.1. Cohort

This study included 508 patients (307 (60%) planned for RARP and 201 (40%) for ORP). The median age was 65 (range: 45–80) years. ORP patients were more often classified with D’Amico high risk (high risk: 37% (75/201) vs. 14% (42/307), *p* < 0.001). RARP patients had higher education (high school; 43% (119/274) vs. 33% (57/172); *p* = 0.02), higher household income (>4000 EUR/month; 36% (99/273) vs. 25% (46/183); *p* = 0.002) and were more likely to be privately insured (32% (99/296) vs. 19% (35/189); *p* < 0.001) (Table 1).

### 3.2. Informational and Decisional Behaviour

**Figure 1:**
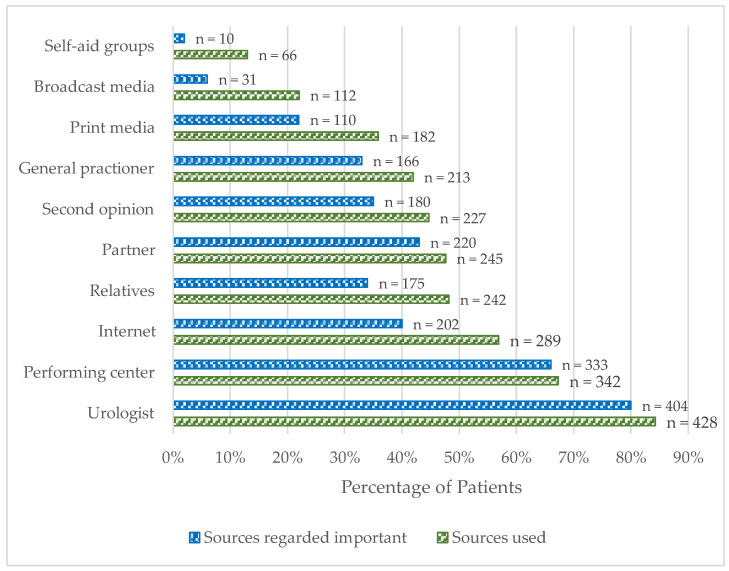
The number of patients rating different sources of information as important.

The top three information sources used by patients before RP were their outpatient urologist (84%, 428/508), the surgery-performing urologic centre (67%, 342/508) and the internet (57%, 289/508). The least important groups were self-aid groups (2%, 10/508) and broadcasting services (6%, 31/508). While the outpatient urologist (80%, 404/508) and the surgery performing urologic centre (66%, 333/508) were also the most important sources of information, the opinion of the partner (43%, 220/508) was rated more often as important than the internet (40%, 202/508) (Figure 1). Online information predominantly stemmed from online forums (33.2%, 96/289), followed by Wikipedia™ (23%, 67/289), online presses (22%, 62/289) and websites of urological societies (21%, 60/289). YouTube (2%, 7/289) and social media (7%, 20/289) were scarcely used.

Compared with ORP patients, RARP patients informed themselves more equally on both procedures (70% (185/264) vs. 49% (82/168); *p* < 0.001). More ORP patients than RARP patients mainly informed themselves about their own procedure (48% (80/168) vs. 26% (69/264); *p* < 0.001).

Compared with ORP patients, RARP patients exhibited more autonomous or informed decision-making behaviour regarding their surgical procedure (47% (133/283) vs. 32% (56/174); *p* < 0.001) (Table 2).

### 3.3. Perception of Outcomes

**Figure 2:**
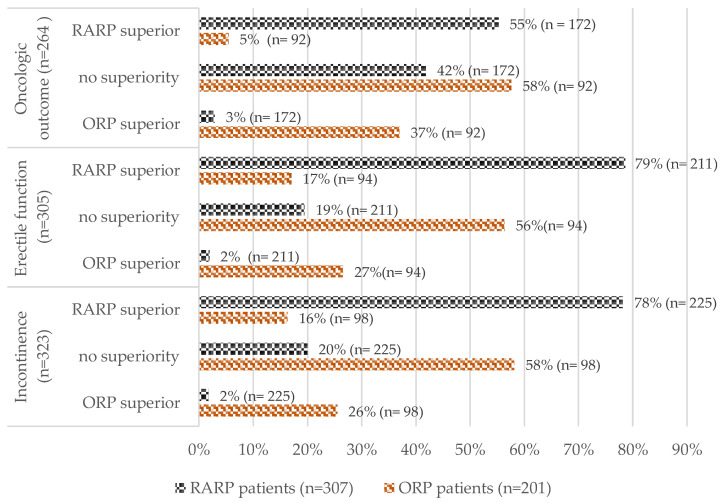
Distribution of preoperative perceptions of oncologic outcomes, postoperative erectile function and incontinence in ORP and RARP patients. ORP = open radical prostatectomy, RARP = robotic-assisted radical prostatectomy.

Compared with ORP patients, RARP patients view RARP rather as superior in terms of curation (55% (95/172) vs. 5% (5/92); *p* < 0.001), postoperative continence (78% (176/225) vs. 16% (16/98); *p* < 0.001) and postoperative erectile function (79% (166/211) vs. 17% (16/94); *p* < 0.001). ORP patients are more convinced of the parity of both procedures in terms of curation (58% (53/92) vs. 42% (72/172); *p* < 0.001), postoperative continence (58% (57/98) vs. 20% (45/225); *p* < 0.001) and postoperative erectile function (56% (53/94) vs. 19% (41/211); *p* < 0.001) (Figure 2 and Supplementary Table S1).

Despite inequal distribution of perceptions, the majority of both RARP and ORP patients perceive RARP to be superior in terms of wound healing (93% (211/228), 66% (67/101); *p* < 0.001) and hospitalization (91% (192/241), 51% (48/94); *p* < 0.001), but not in terms of complications (73% (155/212) vs. 18% (17/97); *p* < 0.001) and reconvalescence (85% (188/253) vs. 43% (40/94); *p* < 0.001) (Supplementary Table S2).

In centres offering both procedures, the belief in RARP’s superiority in curation (49% (70/143) vs. 4% (2/48); *p* < 0.001), continence (76% (143/188) vs. 23% (12/53); *p* < 0.001) and erectile function (78% (138/177) vs. 19% (10/52); *p* < 0.001) remained, although it was slightly less significant compared with centres offering only one technique.

#### Factors for Misperception of Outcomes

Patients perceiving differences in RARP and ORP in terms of curation were older (65.3 vs. 62.6; *p* < 0.001), had lower education (no high school degree: 50% (70/139) vs. 39% (49/125), *p* = 0.02) and were about to receive robot-assisted surgery (RARP: 72% (100/139) vs. 58% (72/125); *p* = 0.02). Patients being treated at a centre offering both procedures were mostly knowledgeable (knowledgeable: 55% (105/191) vs. 45% (86/191); *p* < 0.001), whereas patients treated at a centre offering only one type of approach were mostly unknowing (unknowing: 72% (53/73) vs. 27% (20/73); *p* < 0.001) regarding the information about the procedure. Patients that saw no differences in the two procedures informed themselves on both procedures more equally (equal information on both procedures (78% (97/125) vs. 55% (76/139); *p* < 0.001) (Supplementary Table S2).

In a multivariable analysis, age > 66 years (OR 2.2 (95% CI 1.1–4.6); *p* = 0.02), no high school degree (OR 1.9 (95% CI 1.0–3.6); *p* = 0.047), unbalanced informational behaviour (OR 2.4 (95% CI 1.2–5.1); *p* = 0.02), planned RARP (OR 8.9 (95% CI 3.3–12.8); *p* < 0.001) and being treated at a centre offering only one procedure (OR 3.5 (95% CI 2.0–6.1); *p* < 0.001) were independent risk factors for misconceiving oncological outcomes (Table 3).

## 4. Discussion

This multicentric, prospective study highlights the informational behaviour, decision-making and lay perspectives of patients on the functional and oncological outcomes before RP. As established beforehand, functional and oncological outcomes stand in line with other aspects of comparison between RARP and ORP, including hospitalization, procedure duration, intra- and postoperative complication rates, recovery and cost efficiency. Clear advantages for RARP were seen for intraoperative blood loss, shorter index hospitalisation [3,4,5], total cumulative costs [6] and, assumably, for postoperative complications [3,4,5,6,12], but not in terms of oncological and functional efficacy [3,4,5,10]. Analysing patients’ informational and decisional behaviour and perceptions of advantages is paramount to targeted and weighed patient education.

The patient cohort (*n* = 508) in this study, with a mean age of 65 years and about 62% of patients categorized as intermediate risk, were comparable to other studies [4,5]. The three most self-reported sources of information for patients were outpatient urologists (84%), operating urologic centres (67%) and the internet (57%). However, the sources with the greatest relevance to patients were urologists (80%), operating urologic centres (66%) and the patients’ partners (43%), making personal and subjective sources more important than objective and self-acquired knowledge (Figure 1).

Even though up to 60% of patients generally use online health information [19,22,23], the urologist (84%) and the surgical centre (67%) remain the most important sources of information before the RP. A considerable proportion of patients (57%) use online sources, predominantly online fora and Wikipedia. These findings are consistent with a study demonstrating the influence of online self-aid-fora on therapeutic decisions in up to 29% of PCa patients and highlighting the necessity for high-quality online information [24]. Furthermore, it is interesting to note that trendy internet formats such as social media play a very minor role, in contrast to the frequently used online forums. This may be due to the age group, which is less active on these platforms but may become more involved in the future. Interestingly, video platforms were used extremely rarely (2% ORP; 1% RARP), although these platforms would be ideal for obtaining information, particularly regarding the RARP. The threat of misinformation spread by the video platforms described above is mitigated by their low usage [20].

Finally, the main sources of information were human interactions with consultants at the office or the urologic centre, which mainly influenced patients’ decisions. Only 3% of patients reported making general medical decisions (13% for procedure choice, 26% for centre choice) independently, with a majority of 69% of patients (59% for procedure choice, 54% for centre choice) weighing up their doctors’ opinion as important or more important than their own. RARP patients exhibited more autonomous or informed decision-making behaviour regarding their surgical procedure (47% (133/283) vs. 32% (56/174); *p* < 0.001), leaving more room for informational and decisional influences not induced by medical staff.

As stated above, this study also investigated the outcome of the above informational behaviour, i.e., patients’ preoperative perceptions of objectively equal oncologic and functional outcomes [3,4,5,10] of RARP and ORP. Notably, more RARP patients stated that they would inform themselves equally about both procedures (RARP 60.2% vs. ORP 40.8%; *p* < 0.001). However, RARP patients were more likely to misconceive the oncological and functional equality of the procedures (no superiority: RARP 42% vs. ORP 58%; *p* < 0.001) (Figure 2). In contrast, ORP patients acknowledged being better informed about their own procedure (ORP more on ORP: 48% vs. RARP more on RARP: 26%, *p* < 0.001) but were more convinced of the procedures’ parity (no superiority: RARP 42% vs. ORP 58%; *p* < 0.001). Without objective methods to assess informational behaviour, this effect may be attributed to the study design. The shared majoritarian understanding of both groups regarding RARP’s superiority in terms of wound healing (RARP superior: RARP 93%, ORP 66%) and hospitalization (RARP superior: RARP 91%, ORP 51%) suggests that ORP patients differentiate between the two procedures more correctly, acknowledging the unambiguous advantages of RARP [3,4,5].

The effect of choice-supportive misremembering [25] could have affected the more self-sufficient RARP patients (autonomous or informed decision-making regarding surgical procedure: 47% vs. 32%; *p* < 0.001). Questionnaires were administered when patients were about to provide written consent for their predefined procedures. Another explanation could be the halo effect, where perceptions of one attribute are generalized to other characteristics [26]. This has previously been described in the context of medical products [27]. In this context, it might provide a generalization of the benefits in wound healing, hospitalization and assumingly fewer complications [3,4,5] to oncologic and functional outcomes. Misconception in RARP patients might also be explained by marketing effects. Although more recent data are needed, a 2012 study revealed that online information on procedures is skewed towards favouring RARP on both the websites of manufacturers and third parties [28]. This may lead the more self-sufficient RARP patients to inform themselves in a biased manner, while ORP patients are informed in a more balanced manner by medical personnel.

However, we identified more risk factors for misconceptions of oncological and functional outcomes. Patients with no high school degree (OR 1.9, *p* = 0.047), who are unequally informed (OR 2.4, *p* = 0.02), who are older (OR 2.1, *p* = 0.02) and who are treated at centres offering only one procedure (OR 3.5; *p* < 0.001) may be prone to misconception, and a risk of discrimination is imminent. Elderly individuals seek health information less actively and might be more susceptible to misunderstandings [29]. Improving older patients’ understanding of procedures is achieved best through in-person contact [30]. Our study underlines this. Although online sources are the third most used (57%), they are ranked less important (40%) than personal sources are (Figure 1). As knowledge gained on PCa during consultations does not depend on the patient’s previous education, personal interaction may be an opportunity to address education as a risk factor (OR 1.9, *p* = 0.047). The comprehensive disadvantage of being treated at a centre offering one procedure (OR 3.5, *p* < 0.001) underscores this. However, centres offering both procedures inhouse also need to address differences in procedures and their outcomes individually. An optimal way of doing so would be by presenting internal rates of complications, satisfying outcomes etc. ahead of jointly choosing one procedure. Gaining a better understanding in a self-sufficient manner is difficult, as the reading levels of online sources are too advanced for most patients [31].

This study has several limitations. The questionnaires were completed shortly before the consultation at the centre, when surgery was already scheduled. Therefore, it is possible that participants were primed and thus unequally informed, and a choice-supportive method can lead to misremembering. Nevertheless, both limitations can also explain the patients’ misperception regarding the procedure. Patients may also experience the bias of social desirability, upscaling their role in information gathering and favouring the planned procedure. Since this is the first study to address this topic, there is not yet a validated method for classifying whether a patient is knowledgeable about the procedure options. We have therefore chosen the oncological outcome for this purpose since it is crucial for the patient, and the results show no difference between the two surgical procedures [8,12].

This prospective multicentric study design with centres offering both or only one procedure provides an in-depth overview of the information behaviour and the perceptions of both procedures. This is the first study evaluating this topic, which is especially relevant in countries where patients have the option to choose between the two procedures. Although patients had already been scheduled for surgery when they were interviewed, they still underwent a preoperative interview, during which this misperception could also be addressed. The question is what influence this misperception has on the postoperative judgement of patients. A follow-up survey of this cohort is already planned.

## 5. Conclusions

Compared to ORP patients, RARP patients wrongfully considered their intervention to be more beneficial for good oncological and functional outcomes. In terms of risk factors for this misperception, this may be due to unbalanced sources of information available to patients. Patients at the highest risk for misconceptions were those above 66 years, with low education levels, being treated at a centre offering only one of both procedures. The urologist was the most important source of information, followed by other personal and subjective sources, with the internet ranking third. Urologists and surgical centres must thus be aware of the informational landscape, risk factors and choice-supportive misremembering to address misperceptions, especially regarding RARP, and enable patients to make truly informed decisions.

## Figures and Tables

**Table 1:** Information on the study cohort (*n* = 508).

	All (*n* = 508)	ORP (*n* = 201)	RARP (*n* = 307)	*p* Value
Age	64.8 ± 6.9 65 (45–80)	64.5 ± 6.7 65 (45–77)	65.0 ± 7.0 66 (45–80)	0.5 *
D’Amico classification				
Low risk	78 (15%)	29 (14%)	49 (16%)	**<0.001**
Intermediate risk	313 (62%)	97 (48%)	216 (70%)	
High risk	117 (23%)	75 (37%)	42 (14%)	
Insurance status (*n* = 485)				
Statutory	351 (77%)	154 (81%)	197 (67%)	**<0.001**
Private	134 (29%)	35 (19%)	99 (33%)	
Living area (*n* = 494)				
Rural	239 (48%)	101 (51%)	138 (47%)	0.3
City	255 (52%)	97 (49%)	158 (53%)	
Marital status (*n* = 494)				
not Married	52 (11%)	20 (10%)	32 (11%)	0.9
Married	442 (89%)	176 (90%)	266 (89%)	
Educational degree (*n* = 446)				
None	1 (0%)	1 (1%)	0 (0%)	**0.02**
Lower secondary school	113 (25%)	56 (33%)	57 (21%)	
Secondary school	156 (35%)	58 (34%)	98 (36%)	
High school	176 (39%)	57 (33%)	119 (43%)	
Net income/m in EUR (*n* = 456)				
<1500	35 (8%)	22 (12%)	13 (5%)	**0.002**
1500–4000	276 (61%)	115 (63%)	161 (59%)	
>4000	145 (32%)	46 (25%)	99 (36%)	
Study centre				
1	167 (33%)	44 (22%)	123 (40%)	**<0.001**
2	49 (10%)	0 (0%)	49 (16%)	
3	49 (10%)	49 (24%)	0 (0%)	
4	93 (18%)	28 (14%)	65 (21%)	
5	53 (10%)	13 (6%)	40 (13%)	
6	55 (11%)	55 (27%)	0 (0%)	
7	42 (8%)	12 (6%)	30 (10%)	

Chi-squared test was used for *p* values. * = unconnected *t*-test; ORP = open radical prostatectomy; RARP = robotic-assisted radical prostatectomy. Bold numbers indicate significance at α = 0.05.

**Table 2:** Comparison of the informational and decisional behaviour of RP patients between the ORP and RARP groups (*n* = 508).

	All (*n* = 508)	ORP (*n* = 201)	RARP (*n* = 307)	*p* Value
Information acquisition on RP (*n* = 388)				
More on RARP	75 (17%)	6 (4%)	69 (26%)	**<0.001**
Equally	267 (62%)	82 (49%)	185 (70%)	
More on ORP	90 (21%)	80 (48%)	10 (4%)	
Internet usage for health-related topics (*n* = 490)				
Daily	20 (4%)	8 (4%)	12 (4%)	0.2
1/week	86 (18%)	28 (15%)	58 (20%)	
Less than 1/week	314 (64%)	123 (64%)	191 (64%)	
Not at all	70 (14%)	34 (18%)	36 (12%)	
Online sources used				
Video platforms	7 (1%)	4 (2%)	3 (1%)	n.a.
Social media	20 (4%)	8 (4%)	12 (4%)	
Expert association	60 (12%)	24 (12%)	36 (12%)	
Online press	62 (12%)	20 (10%)	42 (14%)	
Wikipedia	67 (13%)	29 (14%)	38 (12%)	
Online fora	96 (19%)	40 (20%)	56 (18%)	
Others	127 (25%)	55 (27%)	72 (23%)	
None	12 (3%)	6 (3%)	6 (2%)	
Decisional behaviour general (*n* = 476)				
Self-sufficient	12 (3%)	6 (3%)	6 (2%)	0.2
Considering experts’ opinion	140 (29%)	48 (25%)	92 (32%)	
Jointly	289 (61%)	116 (61%)	173 (60%)	
Considering own opinion	31 (7%)	17 (9%)	14 (5%)	
Doctors’ decision	4 (1%)	2 (1%)	2 (1%)	
Decisional behaviour regarding surgical procedure (*n* = 457)				
Self-sufficient	59 (13%)	14 (8%)	45 (16%)	**<0.001**
Considering experts’ opinion	130 (28%)	42 (24%)	88 (31%)	
Jointly	190 (42%)	75 (43%)	115 (41%)	
Considering own opinion	52 (11%)	23 (13%)	29 (10%)	
Doctors’ decision	26 (6%)	20 (11%)	6 (2%)	
Decisional behaviour regarding performing centre (*n* = 482)				
Self-sufficient	127 (26%)	52 (27%)	75 (26%)	0.3
Considering experts’ opinion	96 (20%)	36 (19%)	60 (21%)	
Jointly	168 (35%)	61 (32%)	107 (37%)	
Considering own opinion	42 (9%)	23 (12%)	19 (7%)	
Doctors’ decision	49 (10%)	18 (9%)	31 (11%)	

Chi-squared test was used for *p* values. RP = radical prostatectomy; ORP = open radical prostatectomy; RARP = robotic-assisted radical prostatectomy. Bold numbers indicate significance at α = 0.05. n.a. = not available, as variables are multiple-choice based.

**Table 3:** Multivariable analysis of risk factors influencing patients’ misconception regarding the oncologic inequality between RARP and ORP.

	Univariable Analysis		Multivariable Analysis	
	OR	*p* Value	OR	*p* Value
Old age (66+)	2.0 (1.2–3.3)	**0.007**	2.1 (1.1–3.9)	**0.02**
No high school degree	1.8 (1,1–3.1)	**0.02**	1.9 (1.0–3.6)	**0.047**
Paternalistic/partially paternalistic decisional behaviour	1.2 (0.8–2.0)	0.4	1.5 (0.8–2.8)	0.3
Unbalanced information acquisition	2.9 (1.6–5.1)	**<0.001**	2.4 (1.2–5.1)	**0.02**
RARP patient	1.9 (1.1–3.2)	**0.02**	8.9 (3.3–23.8)	**<0.001**
No choice of procedure at centre	1.7 (1.2–2.4)	**0.002**	3.5 (2.0–6.1)	**<0.001**

OR = odds ratio; ORP = open radical prostatectomy; RARP = robotic-assisted radical prostatectomy. Bold numbers indicate significance at α = 0.05.

## Data Availability

Original data are available upon request from the corresponding author.

## Supplementary Materials

The following supporting information can be downloaded at: https://www.mdpi.com/article/10.3390/cancers17020300/s1, Table S1: Distribution of patients’ preoperative perceptions of postoperative outcomes after RP among patients planned for ORP and RARP. Table S2: Distribution of patient characteristics among patients perceiving procedures (RARP and ORP) as either oncologically unequal or equal.
