# Supplementary material for: Information Behaviour and Knowledge of Patients Before Radical Prostatectomy
Source: Cancers (Basel). 2025 Jan 17;17(2):300. doi: 10.3390/cancers17020300 (PMC11764233; doi:10.3390/cancers17020300)
Supplement: Supplementary file 1 [file cancers-17-00300-s001.zip › cancers-3390530-supplementary.pdf]

Table S1. Distribution of patients' preoperative perceptions of postoperative outcomes after RP among patients planned for ORP and RARP.

	**All (n=508)**	**ORP (n=201)**	**RARP (n=307)**	**p value**
Assessment of incontinence (n=323)				
ORP superior	29 (9%)	25 (26%)	4 (2%)	
No superiority	102 (32%)	57 (58%)	45 (20%)	**<0.001**
RARP superior	192 (59%)	16 (16%)	176 (78%)	
Assessment of erectile function (n=305)				
ORP superior	29 (10%)	25 (27%)	4 (2%)	
No superiority	94 (31%)	53 (56%)	41 (19%)	**<0.001**
RARP superior	182 (60%)	16 (17%)	166 (79%)	
Assessment of oncologic curation (n=264)				
ORP superior	39 (15%)	34 (37%)	5 (3%)	
No superiority	125 (47%)	53 (58%)	72 (42%)	**<0.001**
RARP superior	100 (38%)	5 (5%)	95 (55%)	
Assessment of complications (n=309)				
ORP superior	32 (10%)	28 (29%)	4 (2%)	
No superiority	105 (34%)	52 (54%)	53 (25%)	**<0.001**
RARP superior	172 (56%)	17 (18%)	155 (73%)	
Assessment of postoperative pain (n=310)				
ORP superior	19 (6%)	16 (16%)	3 (1%)	
No superiority	69 (22%)	41 (41%)	28 (13%)	**<0.001**
RARP superior	222 (72%)	43 (43%)	179 (85%)	
Assessment of wound healing (n=329)				
ORP superior	15 (5%)	11 (11%)	4 (2%)	
No superiority	36 (11%)	23 (23%)	13 (6%)	**<0.001**
RARP superior	278 (84%)	67 (66%)	211 (93%)	
Assessment of duration of hospitalization (n=315)				
ORP superior	16 (5%)	12 (13%)	4 (2%)	
No superiority	59 (19%)	34 (36%)	25 (12%)	**<0.001**
RARP superior	240 (76%)	48 (51%)	192 (91%)	
Assessment of duration of catheterization (n=281)				
ORP superior	16 (6%)	14 (16%)	2 (1%)	
No superiority	110 (39%)	51 (57%)	59 (31%)	**<0.001**
RARP superior	155 (55%)	25 (28%)	130 (68%)	
Assessment of reconvalescence (n=316)				
ORP superior	16 (5%)	13 (14%)	3 (1%)	
No superiority	72 (23%)	41 (44%)	31 (14%)	**<0.001**
RARP superior	228 (72%)	40 (43%)	188 (85%)	

RP = Radical prostatectomy, ORP = Open radical prostatectomy, RARP = Robotic assisted radical prostatectomy.

Table S2. Distribution of patient characteristics among patients perceiving procedures (RARP and ORP) as oncologically inequal or equal.

	**All** (n=264)	**Oncological inequality** (n=139)	**Oncological equality** (n=125)	**p value**
Age	64.0 ± 6,6<br>65 (45–77)	65.3 ± 6.2<br>66 (47–77)	62.6 ± 6.8<br>63 (45–75)	**<0.001***
D'Amico classification				
Low risk	50 (19%)	28 (20%)	22 (18%)	0.9
Intermediate risk	165 (62%)	85 (61%)	80 (64%)	
High risk	49 (19%)	26 (19%)	23 (18%)	
Surgical procedure				
ORP	92 (35%)	39 (28%)	53 (42%)	**0.015**
RARP	172 (65%)	100 (72%)	72 (58%)	
Procedural choice offered at centre				
One procedure	73 (28%)	53 (38%)	20 (16%)	**<0.001**
Both procedures	191 (72%)	86 (62%)	105 (84%)	
Living area (n=261)				
Urban	134 (51%)	67 (49%)	67 (54%)	0.4
Countryside	127 (49%)	71 (51%)	56 (46%)	
Insurance status (n=235)				
Statutory	176 (70%)	91 (70%)	85 (69%)	0.9
Private	77 (30%)	39 (30%)	38 (31%)	
Marital status (n=264)				
not Married	25 (9%)	11 (8%)	14 (11%)	0.4
Married	239 (91%)	128 (92%)	111 (89%)	
Educational degree (n=233)				
Lower secondary school	46 (20%)	31 (26%)	15 (13%)	**0.02**
Secondary school	73 (31%)	39 (32%)	34 (30%)	
High school	114 (49%)	50 (42%)	64 (57%)	
Income/m in EUR (n=241)				
<1500	15 (6%)	9 (7%)	6 (5%)	0.2
1500-4000	144 (60%)	81 (64%)	63 (55%)	
>4000	82 (34%)	36 (29%)	46 (40%)	

Decisional behaviour general (n=261)				
Self-sufficient	6 (2%)	2 (1%)	6 (2%)	0.4
Considering expert's opinion	91 (35%)	48 (35%)	91 (35%)	
Jointly	149 (57%)	76 (55%)	149 (57%)	
Considering own opinion	13 (5%)	9 (7%)	13 (5%)	
Expert	2 (1%)	2 (1%)	2 (1%)	
Decisional behaviour on surgical procedure (n=257)				
Self-sufficient	38 (15%)	15 (11%)	23 (19%)	**0.08**
Considering expert's opinion	81 (32%)	45 (33%)	36 (30%)	
Jointly	104 (40%)	54 (39%)	50 (42%)	
Considering own opinion	22 (9%)	17 (12%)	5 (4%)	
Expert	12 (5%)	6 (4%)	6 (5%)	
Decisional behaviour on performing centre (n=260)				
Self-sufficient	71 (27%)	30 (22%)	41 (33%)	0.057
Considering expert's opinion	53 (20%)	24 (18%)	29 (23%)	
Jointly	93 (36%)	53 (39%)	40 (32%)	
Considering own opinion	18 (7%)	11 (8%)	7 (6%)	
Expert	25 (10%)	18 (13%)	7 (6%)	
Internet usage for health-related topics (n=260)				
Daily	14 (5%)	6 (4%)	8 (7%)	0.3
1/week	53 (20%)	32 (23%)	21 (17%)	
Less than 1/week	167 (64%)	83 (60%)	84 (69%)	
Not at all	26 (10%)	17 (12%)	9 (7%)	
Information acquisition on RP (n=251)				
More on RARP	44 (18%)	34 (26%)	10 (8%)	**<0.001**
Equally	173 (69%)	76 (58%)	97 (80%)	
More on ORP	34 (14%)	20 (15%)	14 (12%)	

RP = Radical prostatectomy, ORP = Open radical prostatectomy, RARP = Robotic assisted radical prostatectomy.
